# A Tailored mHealth App for Improving Health and Well-Being Behavioral Transformation in UK Police Workers: Usability Testing via a Mixed Methods Study

**DOI:** 10.2196/42912

**Published:** 2023-08-04

**Authors:** Richa Mehra, Andy Pulman, Huseyin Dogan, Jane Murphy, Fiona Bitters

**Affiliations:** 1 Bournemouth University, Faculty of Science and Technology Bournemouth United Kingdom; 2 Bournemouth University, Faculty of Health and Social Sciences Bournemouth United Kingdom; 3 Hampshire Constabulary Southampton United Kingdom

**Keywords:** nutrition, activity, behavior change, telemedicine, mobile health, police, lifestyle management, management, usability testing, design, build, prototype, testing, survey, interview, development, user center, officer, law enforcement, cop, detective, policeman, policing, mobile phone

## Abstract

**Background:**

When considering the policing environment of 2022, many roles previously in the domain of warranted officers (police officer) are now performed by nonwarranted police staff equivalents. These police staff roles have expanded rapidly into other areas such as investigations, custody, and contact management, which were traditionally seen as police officer functions and put staff under some of the same stresses as police officers. A UK police force requested help in investigating technologies that could be used to improve health and well-being for both officers and staff.

**Objective:**

The aim of this study was to create a health and well-being app for police officers and staff, which considered the unique requirements of the users throughout the designing, building, prototyping, and testing stages.

**Methods:**

This study involved quantitative approaches (demographic web-based survey questions and the System Usability Scale) and qualitative approaches (open web-based survey questions and semistructured interviews). Unsupervised usability testing of a prototype app was undertaken by members (N=48) of the commissioning client using their smartphones. After completing a preregistration application for screening purposes, participants downloaded a trial version of the app. Then, they completed a web-based questionnaire after testing the app for 10 days. A subsample of participants (9/48, 19%) was interviewed. Deductive thematic analysis was undertaken to identify key themes and subthemes.

**Results:**

Data collected during usability testing concerned the 6 domains of the app—food and diet, activity, fluid intake, sleep, good mental health, and financial well-being—and informed the creation of improved design during prototyping. Some usability and design issues and suggestions for improvements were also addressed and implemented—including shift management and catch-up cards—during this cycle of development.

**Conclusions:**

This study highlights the importance of coparticipation with officers and staff across the entire development cycle, to coproduce a human-centered design methodology to enable the development of a considered and user-centered solution. It demonstrates the need for producing a multifunctional tool rather than focusing purely on an individual element for this user group. It also highlights how linking and being able to track optional, personalized elements of health data against one another, cross-referenced to individual shift patterns, might help to inform and provide users with a chance for reflection and therefore influence behavior change.

## Introduction

Police officers can experience difficulties when managing health and well-being as a result of working long, unsocial hours in a highly pressurized environment [1,2]. Those working in the police face different health challenges from those in the general population, as their job might feature unusual working hours and alternating shift patterns [3]. Police officers have a great chance of being overweight and obese and are at risk of long-term conditions such as cardiovascular disease [4] and cancer [5] compared with the average population. Risk factors are heightened by working in a highly stressful environment, increasing the likelihood or the severity of these issues [6,7]. Compared with the average person, an officer is more likely to experience stress owing to exposure to dangerous situations and traumatic events [8]. Stress can have multiple knock-on effects including insomnia, fatigue, and poor concentration—all of which make performing the job harder than it already is and add to the original problem [9].

When considering the policing environment of 2022, many roles previously in the domain of warranted officers are now performed by nonwarranted police staff equivalents. These police staff roles have expanded rapidly into other areas such as investigations, custody, and contact management, which were traditionally seen as police officer functions and put staff under some of the same stresses as police officers. Officers and staff can also be affected by mental health conditions such as posttraumatic stress disorder, depression, and alcohol abuse [10]. Physical risks are also an issue with increased danger of long-term health problems such as back pain [11,12]. Studies during the COVID-19 pandemic also suggest that stress levels of policing have increased during this period and have affected officer resiliency [13,14].

Although some risk factors relating to health and an individual’s risk of certain health issues are nonmodifiable such as age and genetics, modifiable lifestyle factors can be self-managed by individuals to reduce the risk. Currently, there is a large number of health and fitness solutions available on multiple platforms, but none are tailored to specific issues that the police force face or are configurable for the types of routines and working patterns they regularly encounter [15]. Behavior change is made more difficult given the variable working environments that officers and staff can encounter [1,2].

A UK police force approached the project team and requested help in investigating new technologies that could be used to improve health and well-being.

## Methods

### Preliminary Design Cycle

Preparatory work coincided with the first period of the UK lockdown for COVID-19. Web-based surveys were used to gather feedback and information from the user base. Survey data were gathered from 213 participants of the UK commissioning force [15]. Data highlighted that a multifunctional tool would be more beneficial than focusing on a single element. Key features and 4 domains were identified for initial app coverage. In order of importance—prioritized by participant response—these were: food and diet (76/213, 35.6%), activity (68/213, 31.9%), sleep (27/213, 12.6%), and fluid intake (27/213, 12.6%). Participants also identified a need for the new app to consider that a user was on shift—this is important because many issues and problems with elements of their health and well-being involved shift work. For example, shift work and fatigue have been shown to interfere with sleep and impair cognitive function [16,17].

### Secondary Design Cycle

Initial requirements were categorized using the MoSCoW (Must Have, Should Have, Could Have, Won’t Have) framework [18], with findings from the web-based questionnaire and client meetings informing initial draft requirements. Paper designs were sketched leading to the creation of low-fidelity wireframes [15]. These were shown to the client for feedback before high-fidelity designs were created. These were then shown to interviewees during semistructured interviews (n=10), to gather feedback about requirements and preferences for the app. On the basis of their feedback, the second set of design prototypes was created. A good mental health section was added as the fifth domain, including the ability to complete an optional mood diary and track and set alcohol goals. Given the emerging evidence about the impacts of COVID-19 on police staff’s mental well-being before [10,19] and during the pandemic [13,14], the suggested expansion to incorporate a section regarding mental health and well-being seemed to be a valuable addition, especially in light of potential challenges broaching these topics within a culture where discussing mental health difficulties have sometimes been viewed as an undesirable discussion topic [20]. Considerations and concerns of financial nature [21-23] inspired the inclusion of the sixth domain, to cover holistic wellness. It was highlighted that information regarding financial planning, pension policies, and budget and saving advice would be helpful and give control to those who were nearing retirement. Overall wellness is something achieved by taking care of mental, physical, and financial well-being. The suggestion to include financial well-being was incorporated late into the second development cycle.

### Testing Version

Further development using the revised high-fidelity designs as an initial foundation was undertaken with an external developer to produce a prototype for pilot testing. The app—created in Android and Apple iPhone Operating System—was developed using an agile approach. Coding was conducted between October 2021 and April 2022. Several revised versions were developed to include all MoSCoW requirements identified as “must” and “should” have [18]. The process of developing the app to this point was highly iterative and agile.

### Study Design

The purpose of this study was to create a health and well-being app for police officers and staff, which considered the unique requirements of the users throughout the designing, building, prototyping, and testing stages and helped the researchers understand the users and their requirements. The study involved quantitative approaches (demographic web-based survey questions and the System Usability Scale [SUS] [24]) and qualitative approaches (open web-based survey questions and semistructured interviews). We used a human-centered approach to optimize the understanding and accommodate the perspectives of potential users [25]. The methods chosen were considered with the participants in mind—those that could be completed in 1 session, where time could be set aside when they were free. The methods used in this study acknowledged and catered to the logistical and operational pressures that the participants might be under, while allowing them to provide the project team with detailed feedback.

### Participants, Recruitment, and Consent

Participants were recruited via a gatekeeper from the commissioning client. The gatekeeper’s role was to initiate communication between the researcher and police officers and staff who wished to participate—via distribution of study literature—without compromising anonymity or affecting the veracity of web-based responses. Participation was voluntary, and participants remained anonymous to both the gatekeeper and the organization. Consent for completing the web-based survey was requested before allowing participants to proceed. Before each interview began, the researcher answered any questions they had, checked if they were willing to be audio recorded, and explained the consent process before recording began.

### Procedures and Measures

**Figure 1:**
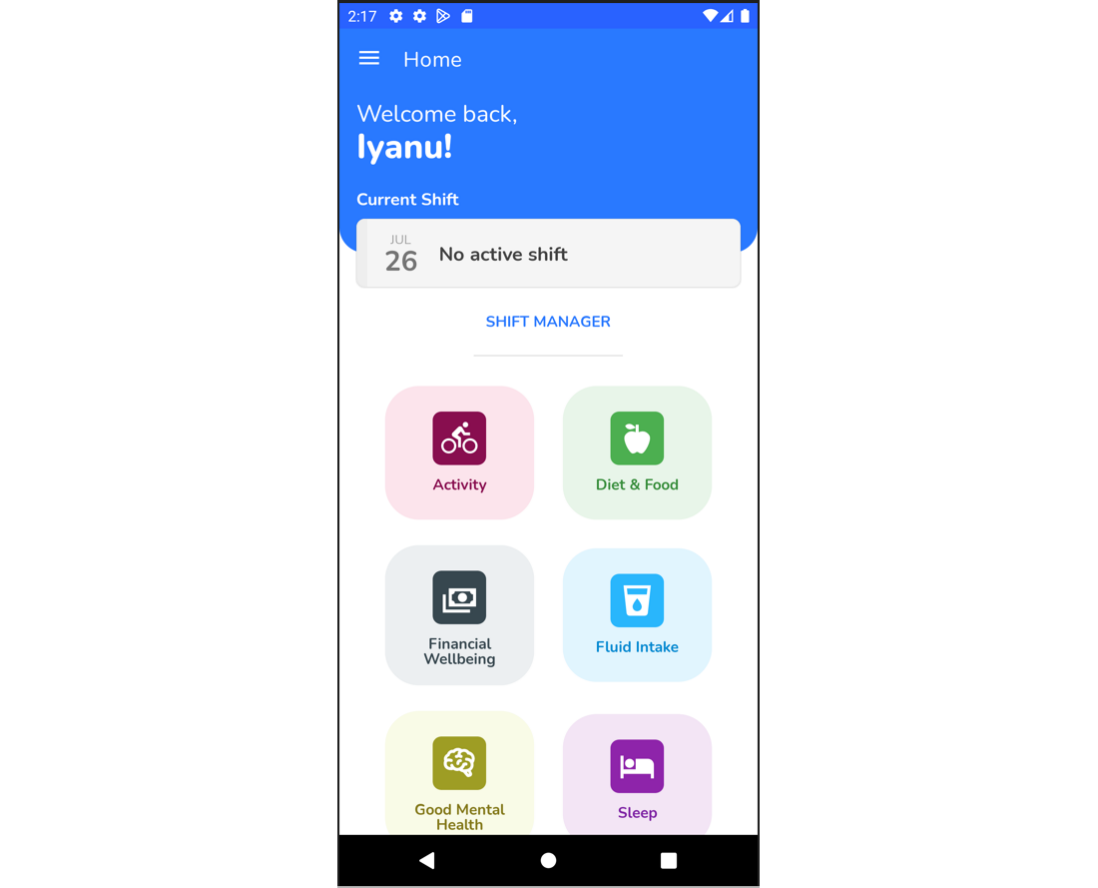
Screenshot of home screen.

This study involved unsupervised usability testing of a prototype app by members of the commissioning client using their smartphones. The version of the app supplied for testing contained draft versions of all 6 sectional domains previously identified—food and diet, activity, sleep, fluid intake, good mental health, and financial well-being—accessible from the home screen (Figure 1). An additional feature provided the opportunity to add forthcoming shift pattern details to the app, which could then be cross-referenced with other parts of the app. The testing version concentrated on implementing basic operational functionality, which could then be expanded or altered as required based on user feedback.

Initially, an email was sent to police officers and staff describing the study and asking about their interest. After completing a preregistration application for screening purposes, participants were emailed a link to allow them to download the trial app. A task guide was also sent, giving them 10 days of time for testing. The guide suggested task scenarios of different complexity levels and covered the core functionalities of the app: shift manager, fluid intake, sleep, mood diary, alcohol consumption tracker, financial well-being, and activity. Participants were then asked to complete a web-based questionnaire after 10 days of testing. Demographic details, information about use of the app, likes and dislikes, and feedback about specific features were collected through open-ended questions. The questionnaire was completed by 48 participants.

A subsample of participants (9/48, 19%) who provided consent to a follow-up telephone interview via a questionnaire subsection were interviewed after they had completed the given tasks. Topic guides were used for the semistructured interviews, to ensure that areas of interest (such as aspects related to delivery mode and format) were covered, while still allowing flexibility. Participants were asked several follow-up questions about the content, design, and functionalities of the app and to rate their experience. Throughout the session, the interviewer encouraged the participants to speak aloud about their actions, which helped to understand the emotions of the user while using the functionalities of the app. They were encouraged to share the issues faced while performing a task or give suggestions for improving the design. Interviews were audio recorded and transcribed verbatim. Notetaking was also used to collect data during the usability sessions.

### Sample Size

As the intended audience for the final product was a UK police force, the work force value supplied by the client organization was rounded to the nearest 1000 and used to calculate an ideal sample size. The value used for the work force was 5000 [26]. The actual number of participants was 48, which was above the lower threshold for an acceptable number for the sample size. With this number of participants and the estimated population size*,* there is a 95% confidence level, with a final margin of error of 14%. This sample size was considered to be adequate because of restrictions in accessing participants—for downloading and testing the app, completing the survey, and possibly participating in an interview—owing to logistical and operational pressures.

### Analysis

Recordings were transcribed and thematically analyzed using a deductive approach that focused on the domains covered in the topic guide (focusing on design, functionalities, and content). A generic qualitative approach to thematic analysis was used [27], with interresearcher interpretation.

### Ethics Approval

Ethics approval was obtained from Bournemouth University (39100).

## Results

### Participant Characteristics

We recruited 33% (16/48) male and 67% (32/48) female participants. The “other” or “prefer not to say” option was also included within the survey question—no responses were received. (Table 1).

**Table 1:** Self-reported descriptions about participants (N=48).

Characteristic			Participants
**Sex, n (%)**			
	Male		16 (33)
	Female		32 (67)
**Age group (years), n (%)**			
	<18		0 (0)
	18-30		4 (8)
	31-40		20 (42)
	41-50		15 (31)
	51-60		8 (17)
	61-70		1 (2)
	>70		0 (0)
**Job role, n (%)**			
	Police officer		20 (42)
	Police worker		28 (58)
**Shift worker, n (%)**			
	Yes		40 (83)
	No		8 (17)
**System Usability Scale**			
	Score, median (range)		73.8 (30-100)
	Score, mean (SD; 95% CI)		70.9 (14.1; 66.9-74.9)
	**Adjective rating (Sauro-Lewis), n (%)**		
		A or A+	11 (23)
		B	13 (27)
		C	12 (25)
		D	5 (10)
		E	7 (15)

### Summary Statistics for the SUS

**Figure 2:**
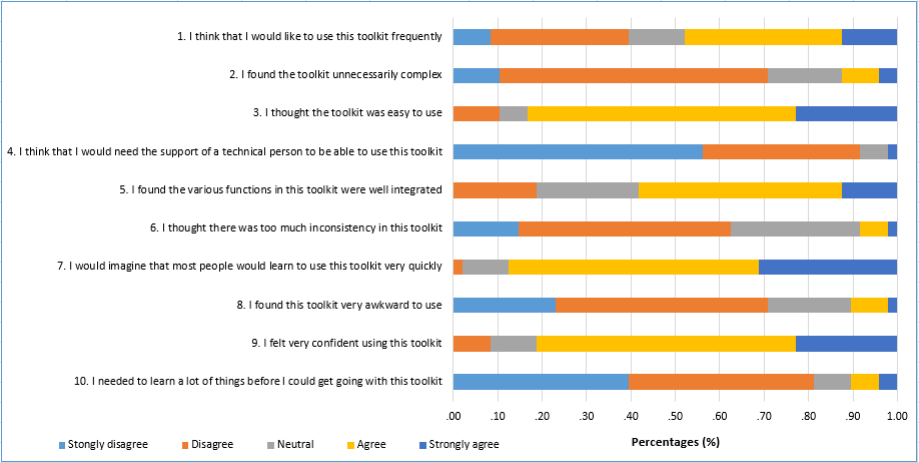
Frequency of responses for the System Usability Scale items.

Summary statistics for the SUS scores are presented in Table 1. Frequencies of ratings for each of the 10 SUS items are shown in Figure 2. Overall, the median SUS score for the toolkit was 73.8 and the mean was 70.9 (SD 14.1; range 30-100; 95% CI 66.9-74.9). This equates to an adjective rating of “OK” [24] and a “C” (41st to 59th percentile range) on the Sauro-Lewis curved grading scale [28]. Most participants (23/48, 48%) thought they “would like to use this toolkit frequently” (the version of the prototype they tested; SUS question 1). Most (40/48, 83%) considered the toolkit “easy to use” (SUS question 3), with 88% (42/48) believing that most people would “learn to use it quickly” (SUS question 7). More than half (28/48, 58%) considered the toolkit’s functionality to be well integrated (SUS question 5).

### App Feedback—Domains

Feedback from survey data and interviews—encompassing themes of design, functionality, and content—was organized into the previously mapped areas of the app. Suggestions made for additional functionality were considered and included any relevant feedback elicited during interviews.

#### Food and Diet

Police personnel wanted to have a tracker on the app to monitor the food that they consumed daily. Most of the users interviewed (6/9, 67%) found the supporting information under this section to be quite relevant and informative. During interviews, 22% (2/9) of the participants mentioned that they liked the detailed information provided about immunity support:

*One really good to see the immunity support page.not a lot of people know how different foods can affect you and stuff.* [Participant 527]

Additional suggestions were the ability to view a summary of how changing shifts could affect healthy food consumption.

#### Activity

**Figure 3:**
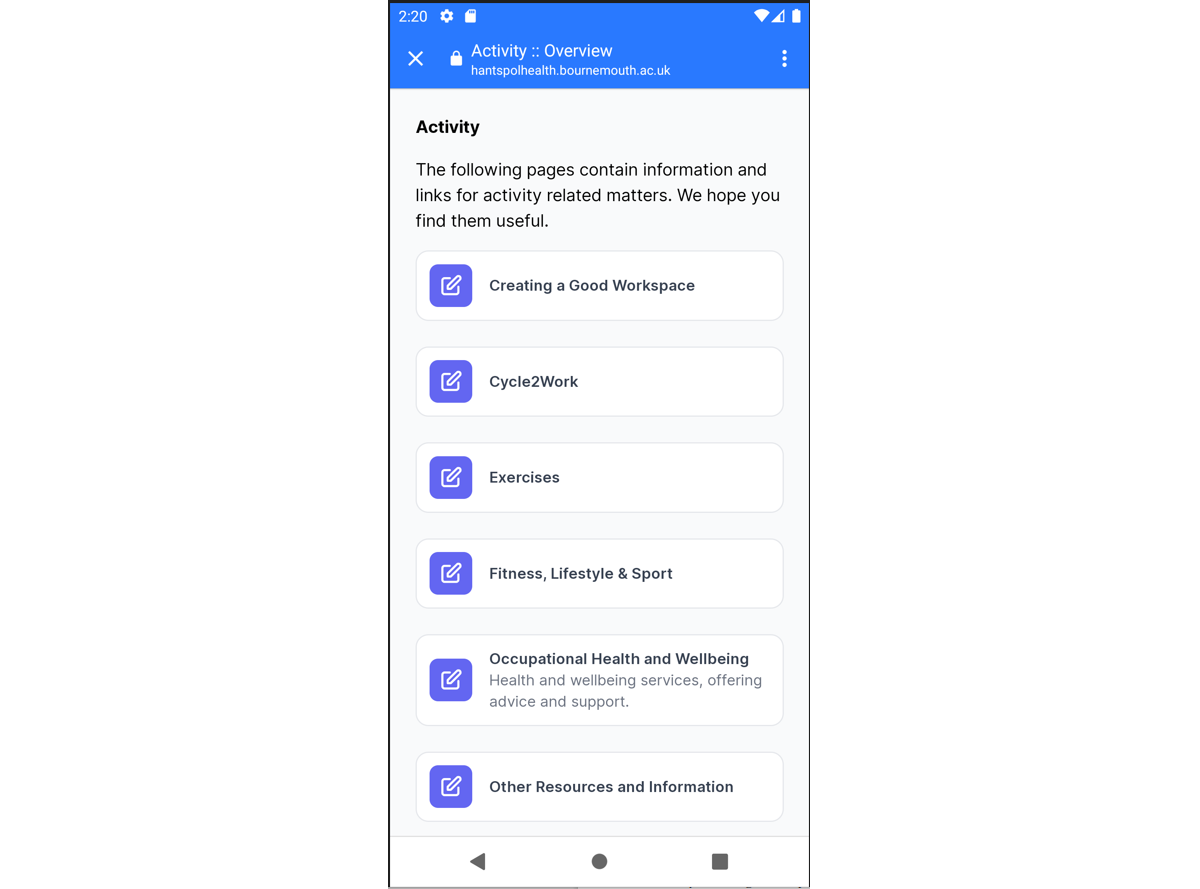
Screenshot of activity section.

The activity section (Figure 3) is currently incomplete without a fully developed tracker, and information provision is not going to help in maintaining good fitness levels in isolation. However, most participants (7/9, 78%) thought that the information provided was relevant, helpful, and comprehensive. They especially mentioned that “Cycle2Work”—a UK government tax exemption scheme, introduced to promote healthy journeys to work—and other sections were good to have on an app as they enabled easy access to all the required wellness material from home. Older participants appreciated the information displayed on posters and found it easy to read. During interviews, participants also mentioned that they would like to track all the physical activities they were doing, including linking the app to their step counter:

Ability to linking with step counter. Idea is to have everything at one place.

Participant 517

Another suggestion was the ability to share information within teams to act as a motivator:

It definitely doesn’t need to rival Strava or MyFitnessPal to be useful but in the activity section a means of tracking exercise [only basic - not pace, incline etc] to perhaps make teams or share stats within or across shifts. To set up a Contact Management leader board of which shift is taking the most steps for example.

Participant 617

#### Fluid Intake

**Figure 4:**
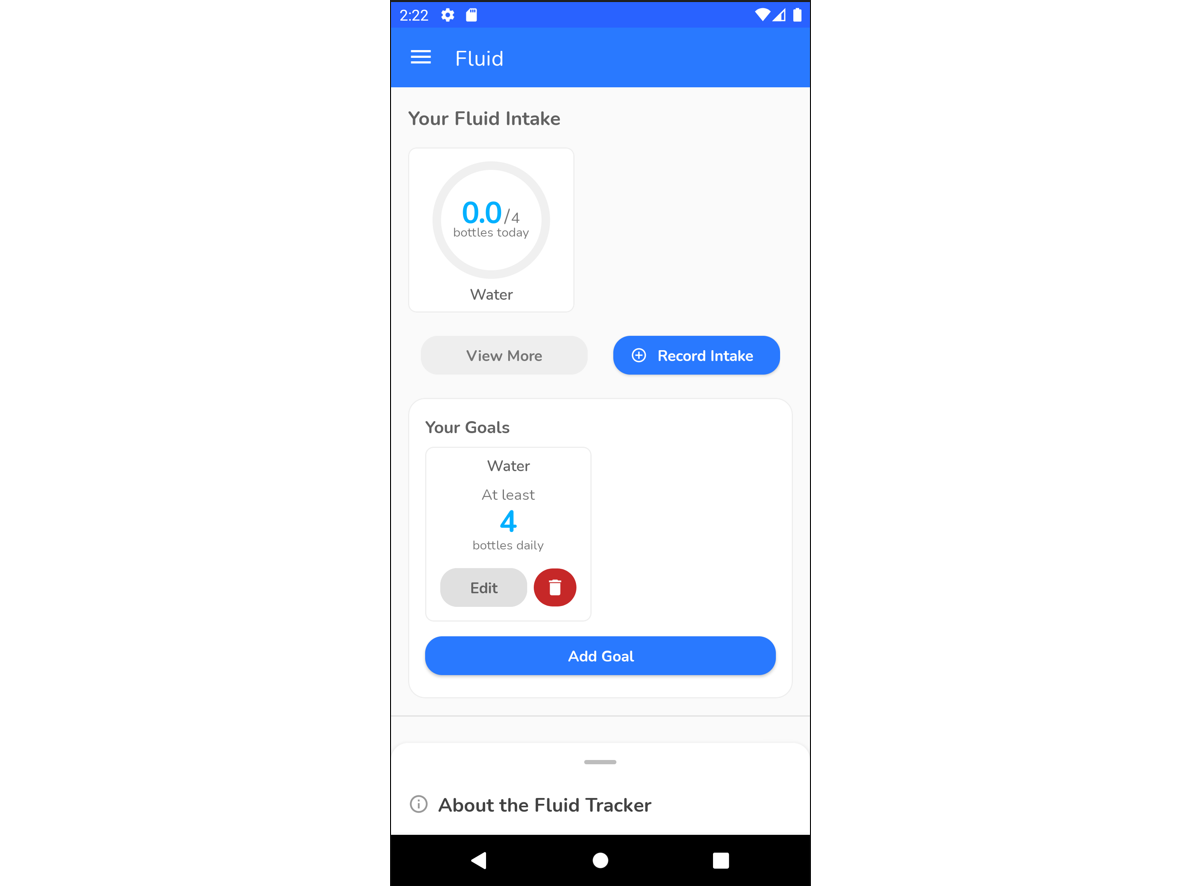
Screenshot of fluid intake section.

Feedback regarding the fluid intake section (Figure 4) included the calendar feature associated with the trackers not working properly to input fluid and alcohol; it displayed units of fluid or alcohol drunk on the current day, even if the entry was made for previous days. Overall, 22% (2/9) of the interviewed participants did not find the fluid tracker to be user-friendly and requested a more detailed help section to guide the user through this section. Participants wanted to have reminders set for fluid intake and record old entries for those that might have been missed. However, catch-up cards (refer to the *App Feedback—Other Features* section) that give reminders to drink water should ideally direct users immediately to the fluid intake tracker for a smooth transition. During interviews, 44% (4/9) of the participants mentioned the desirability of viewing a summary:

So you were making entries every day, but if one day if you would like to see the pattern of your fluid intake, you should get the kind of graph or some.

Participant 527

#### Sleep

Interviewed participants (8/9, 89%) described the information provided about sleep as useful and quite vast. There were some comments about the consistent formatting of the content, and users gave their preference in the web-based survey to style as type 1, which was not only easy on the eyes but also more user-friendly.

During interviews, 56% (5/9) of the participants recommended having the ability to log sleep:

...Load loads of nice information. Yeah, but it would have been nice to have been able to have as a log.

Participant 530

#### Good Mental Health

Alcohol tracker, mood diary, employee assistance information, and recognizing stress were some of the features that the participants tested. Only some could test all the subsections during the testing period. Regarding the alcohol tracker, some participants (2/9, 22%) did not expect this tracker to be located under this section. For them, it made more sense if it was moved to the fluid intake section. The tracker offers the ability to track how many units of alcohol a user has drunk that day, and in addition to this feature, interviewed participants (4/9, 44%) wanted to see an overall summary in relation to their shift, to understand the alcohol consumption and success in meeting the goal.

The interviewed participants (4/9, 44%) appreciated the National Health Service (NHS) information on feelings and symptoms that can be common with mental health concerns, linked from within the app to recognize the level of stress [29], because for some job roles that required working on the front line, operating at unsocial hours and occasionally experiencing traumatic events increased stress levels. Low mood is an indicator of poor mental health [30], and it is possible to track and compare with the previous days using this feature. Participants mentioned that the mood diary was something they would like to use in the future, as it helped to track changes in mood daily and compare them with previous days. It was viewed by some as being extremely important to keep checking this aspect of their health:

And you can kind of almost pull off reports. Maybe it’s a PDF report or something that shows that actually they may be able to identify that on those two days after a set of three lates. They’re moods quite low or their fluid intake’s really bad or do you know what I mean? So that people may utilize the app more...

Participant 532

However, there was also some apprehension noted in using the mood diary. Concerns were whether the data might be monitored by senior management and that they might be viewed differently as a result.

#### Financial Well-Being

Participants liked the amount and types of information included in this section:

I there was some really useful stuff on there that was sort of flicking through with them, particularly around sort of the money saving. I read a lot about the police mutual bit and that was really good. And again gave me more stuff to think about and it’s got all the links for stuff that I use anyway, like the HPL bit and the blue Light card section and all that sort of stuff.

Participant 517

Having all the information easily available on the app was appreciated. Participants wanted to see the *search* function when the reading list was long and screen scrolling was required.

### App Feedback—Other Features

#### Dashboard and Visualization

Survey respondents (27/48, 56%) appreciated the clean look of the app and the fact that the home page was divided into subheadings, which not only eased navigation but also made it easy to use:

...Layout was quite visually appealing so the subheadings. So, you kind of, you knew what you were gonna get when you went into those subheadings, they were specific, they weren’t vague.

Participant 523

They emphasized that they would like to see the summary dashboard in a graphical form for each domain in correlation with the shifts attended. It is always helpful to see the progress made to maintain the motivation levels high, which is crucial during self-monitoring.

#### Shift Manager

**Figure 5:**
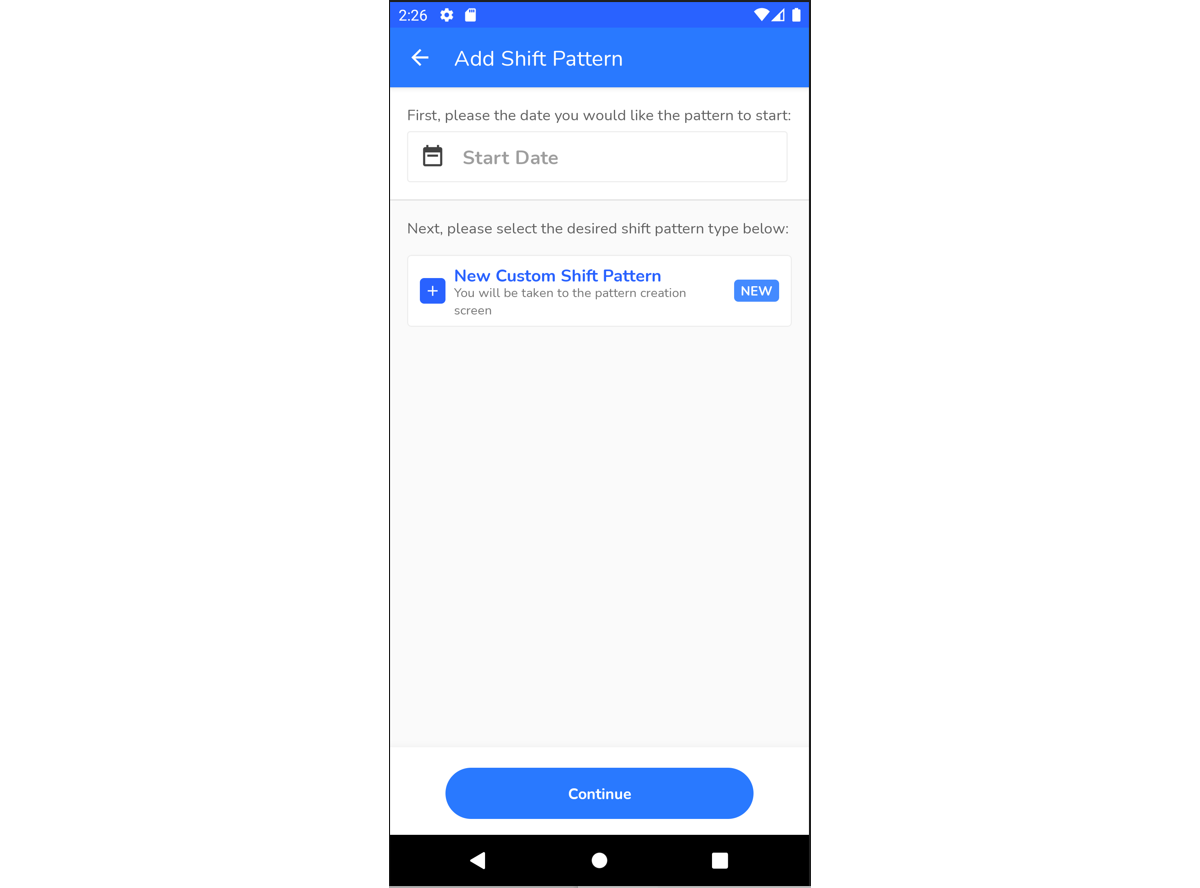
Screenshot of shift manager section.

The shift manager (Figure 5) received positive feedback. A few different iterations were trialed to make this feature more user-friendly. The most common officer and staff shifts were made available to users, so that they could choose the one that suited their pattern best. In addition, the user can make modifications and customize the shift patterns if there were any last-minute changes.

However, there were some challenges. Some participants (18/48, 38%) suggested that the ability to view and edit the entire pattern of the shift in one go would save them manual input time. They also suggested replicating the edited pattern for future use:

I couldn’t figure out a way to put in like a six week shift pattern and then repeat it. I could only figure out how to do it manually day by day. you could do like a six week Pan and start it on a specific date and then it repeats itself, then obviously I could do it that way and it’ll be a lot easier.

Participant 517

#### Goal Setting and Catch-Up Cards

Participants mentioned that the goal-setting feature was useful when using it within the fluid intake section. There were suggestions to include this feature in other domains.

**Figure 6:**
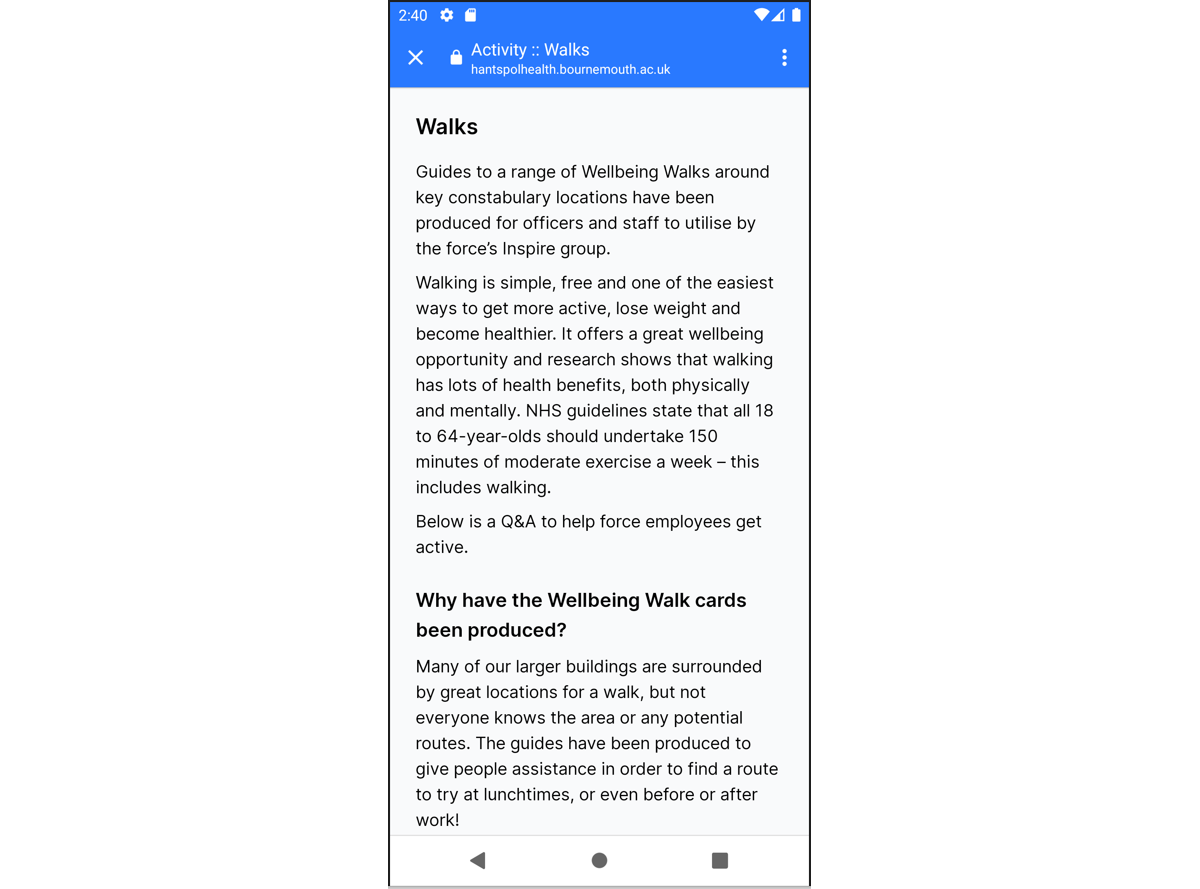
Screenshot of catch-up card - wellbeing walks.

In the test version, a new functionality—catch-up cards (Figure 6)—was introduced in association with shift management improvements. These cards help to remind the users about certain tasks and popped up based on the shift chosen. Participants wanted to use catch-up cards as reminders to meet the set goals. The ability to change the frequency of catch-up cards on an individual basis was something that participants wanted to see in a future release. To have prompts to make increments in their goals was another area identified in which the app could be improved. In addition, if the cards were linked to trackers, they could work as reminders to drink more water or perform some more exercise.

Some would have liked to see the catch-up cards made more interactive, to increase the usability of the cards. In the survey data, 33% (16/48) of the participants could not test this functionality because they were not able to locate it within the app once these were missed as notifications. Users were not able to search for them again if the cards for that day had already been opened and seen once.

Many participants (16/48, 33%) could not understand the timing pattern of the card—at what time of the day cards appeared—and were unable to find the cards later in the app. Notifications were designed to appear 1 hour before the end of a scheduled shift and at 5 PM on a rest day. Currently, the cards are populated to enable appropriate notifications for the shift. For example, during a night shift, it might be focused on sleep, hydration, and relaxation. On a rest day, it might be focused on financial savings and family activity ideas.

## Discussion

### Principal Findings

#### Overview

As our previous study has highlighted, there are currently a number of health and fitness solutions available on multiple platforms in use by police officers and staff [15]. Notably, most apps focus on a particular element of health and well-being. They have not been designed to address the specific issues that police officers and staff face, nor are they configurable for the types of routines and working patterns that the users regularly encounter [31]. Recent literature reviews—such as a 2020 review of studies of gamification and mobile health (mHealth) apps for emergency service personnel (ESP) and police officers across 6 major databases—have highlighted a lack of literature in this area for these groups [32].

To meet the study objectives, where possible, user suggestions were implemented for improvements during the development process. The current app is designed to focus on the individual domains that comprise overall wellness—physical, mental, and financial well-being—of police personnel. Consideration of the shifts undertaken was at its center, as shift work patterns make tracking more difficult for this profession. Overall, feedback about the app was positive based on the SUS score received. Users particularly liked the shift manager, mood diary, trackers to log fluid intake and alcohol consumption, and the relevant supporting information made available for easy access. Approximately half (24/48, 50%) of the users thought that the app was easy to use, and this was considered to be the main reason for liking the app. However, there are still various improvements that need to be actioned. Some of these are improvements in shift manager, giving the ability to create bespoke shift patterns, functioning of the calendar feature of the existing trackers, and display of information to make it easy on the eye. Moreover, there are additional requests by participants to add new features to the app such as trackers to record activity and food intake and reminders to prompt them regarding fluid intake, sleep, and taking rest breaks.

#### Food and Diet

Police workers have great risk of being overweight or obese and risk of developing long-term health conditions [4]. Some of this risk is attributed to poor-quality diet (high in fat, sugar, and salt)—owing to the demands of shift work and the additional occupational stressors associated with police work modifying their relationship with food and unhealthy diet [33]. Police workers might not be able to plan where they might be at any point in a working day owing to the nature of the job, leading to disparate meals, few or small breakfasts, late mealtimes, and increased caloric intake at night [34]. This might also encourage them to make lifestyle choices based on whatever is easiest—for example, getting fast food on the go—rather than preparing healthy food in advance. Interestingly, a recent study by Kosmadopolous et al [34] observed that police officers had great intake of energy from fat and saturated fat during rest days and morning shifts than during evening or night shifts. However, the overall proportions of dietary macronutrients (fat, carbohydrate, and protein) did not significantly differ each day. Kosmadopolous et al [34] observed a series of dietary patterns that implicated the time at which food was consumed, rather than quantity or composition as the differentiating nutritional factor, which might affect metabolic health during shifts.

Working in shifts makes it difficult for police officers and staff to successfully adhere to and sustain healthy lifestyles in the long term. Participants suggested enhanced functionality regarding the ability to view a summary of how changing shifts could have an impact on the consumption of healthy food, and they were also in favor of using only 1 app to monitor all aspects of their health rather than many different ones. There are a number of existing dietary, nutritional, and food information apps available—such as MyFitnessPal [35]—but none of them successfully align completely with the lifestyle, fluctuating shift patterns, and demands of police work [1,2].

#### Activity

NHS guidelines in the United Kingdom [36] recommend that people should be performing some type of physical activity every day. Recent studies in this area involving 2 UK police forces have included a physical activity study using wearables, which used a combination of a Fitbit activity monitor and the “Bupa Boost” smartphone app to promote physical activity and reduce sedentary behavior in police officers [37,38]. Specifically targeted apps—focusing on cycling, running, or a combination of both—are not easily configurable for other activities such as swimming, owing to their design architecture. Buckingham et al [37] noted that there were large individual differences in preferences and perceived impact of the individual and social components of their intervention. These appeared to be owing to personal preferences and personality differences, rather than being associated with any identifiable characteristics, but they highlight how important personalization and tailoring are when considering activity and sedentary behaviors. The study [37] also emphasized that the targeted user group had accepted mHealth technology and found it extremely useful in improving physical health.

Notably, other existing technologies available for use, such as Police Fitness [39], which prepares individuals to pass the initial job entrance fitness exam or their annual fitness check, only concentrate on a particular aspect of fitness. Moreover, they do not naturally integrate with fluctuating shift patterns. The ability to track any kind of physical activity and to see a summary of the impact of shifts on activity levels was an aspect that participants would particularly like to see in a future iteration. Catch-up cards reminding them to participate in occasional physical activity (according to the scheduled shift) were something they considered would make a difference in their wellness journey.

#### Sleep

Among a group of US police officers, it was noted by researchers [40] that sleep disorders were common and significantly associated with increased risk of self-reported adverse health, performance, and safety outcomes. In a more recent study by Fekedulegn et al [41], which examined the association of shift work with sleep quality in police officers, the overall prevalence of poor sleep quality was 54%; 44% for the day shift, 60% for the afternoon shift, and 69% for the night shift. The study concluded that night and evening work schedules were associated with elevated prevalence of poor sleep quality among police officers.

For participants who worked shifts, maintaining regular sleep patterns was not always possible. Owing to the varied times they needed to go to bed and wake up, participants wanted an app that did not rely on standard workdays and the assumption that they would not be working on weekends and, importantly, also allowed them to integrate other features, such as similarly varying mealtimes when on shift and prompting reminders for relaxation before rest periods [15]. After testing the app, the participants expressed a desire to see a summary screen to help with understanding the impact of their shifts upon their sleep in helping to maintain overall wellness. For example, by logging sleep patterns and then comparing and contrasting the types of mood after particular shift patterns.

#### Fluid Intake

Police officers and staff in frontline roles can sometimes find it difficult to maintain hydration status when on duty. Consequently, the effects of dehydration—fatigue, headaches, and irritability—can lead to loss of productivity while working [42]. This has led to campaigns to raise awareness such as the Take-a-Sip campaign—launched with funding from Police Care UK [42]. The NHS guidelines in the United Kingdom [43] recommend drinking between 6 and 8 glasses of fluid per day. Water; low-fat milk; and sugar-free drinks, including tea and coffee, all count toward this total, highlighting that intake does not necessarily need to be solely focused on water intake, which some existing apps might choose to focus on.

Participants mentioned that the goal-setting feature was useful when using it within the fluid intake section. There were suggestions to include this feature in other domains. In the long term, it is intended that, as recommended in the initial study [15], this feature, once finalized programmatically, is replicated and incorporated into other domains.

#### Good Mental Health

In terms of mHealth apps already targeting the mental well-being of police organizations, a UK-based mobile app—Backup Buddy—allows police officers in participating forces to informally view static audio and visual information and signposted support options about common mental health issues [44]. At the end of 2020, Thrive—a mental health and well-being app—was made available to 3500 officers and staff across West Staffordshire, with it also being made available to friends and family if required [45]. In addition, in the United Kingdom, the College of Policing recently conducted a randomized controlled trial, giving 1337 police officers in 5 forces access to either Headspace (a mobile mindfulness app) or Mindfit Cop (a web-based mindfulness resource). This study found that both resources improved well-being, life satisfaction, resilience, and performance compared with the control group. The authors concluded that the trial was sufficiently robust to provide evidence of well-being benefits [30]. A research study is also being conducted by the Police Federation of England and Wales to better understand police officer experiences of using the 87% mental well-being app. This app is designed to support employee well-being strategies [46].

By adding the good mental health section to the designs, a mood diary and the sleep tracker were made available to users to track personalized elements of mood. Integration of the mood diary with the other sections of the app—such as hours of sleep recorded, exercise performed, skipped or eaten meals, and fluid intake—cross-referenced to individual shift patterns in the future would help to inform and provide users with a chance for reflection and therefore influence behavior change. In the long term, this component also has the potential to align more closely with national support services such as the National Police Wellbeing Service [47,48].

However, positives must be viewed alongside concerns from participants that were highlighted in this phase of testing regarding who would be able to access and make assumptions about the data they added. During testing, we were able to confirm that the data were confidential and that no one had access to personal information. This clarification was made to users to encourage use and allay participant concerns regarding how their managers might monitor and subsequently view some of the data entered. It has been noted that individuals in this profession feel reluctant to ask for help for themselves or to discuss their mental health with others. Historically, the police subculture has consisted of values involving masculinity, independence, and emotional control [20]. Such values may make it difficult for many police officers and staff to express emotion or seek mental health treatment, which places them at a disadvantage because internalizing their feelings might reflect in work performance [20]. In such a scenario, it becomes more critical for people in this profession to identify stress early, before it causes additional damage to their mental and physical health. The situation has been exacerbated during and after COVID-19, creating additional uncertainty and increasing the likelihood of stressful situations occurring [13,14].

#### Financial Well-Being

A 2006 study of Taiwanese police officers to assess the quality of life and prevalence of depression in police officers grouped together economic stressors including loans for a house or car, insufficient family income, and debt. According to their results, 52.2% (405/776) of male officers had economic stressors, whereas 30% (17/56) of female officers had economic stressors [21].

Compared with other professions, police officers and staff have different retirement policies; this means that they retire at a younger age than civilians and have more chance of experiencing negative impacts as they move toward retirement [22]. An Italian study found that retirees who were financially well-off were less likely to experience declining health when compared with those who were not [23]. Participants particularly welcomed the inclusion of a financial domain alongside the other aspects of the app, as it gave them good advice and ideas about the pension scheme offered, approaches to saving, and budget planning.

### Shift Manager

Regarding activity, perceived pressure of work and organizational culture appear to be sturdy barriers to reducing sedentary time [49]. Previous studies have highlighted that there was a sense that high workload had resulted in working through breaks and during personal time becoming the norm [50]. Police staff has a mandated lawful requirement to take a break in their shift for which they are not paid, whereas police officers are paid for the full shift. This can mean that when operationally necessary, they work through breaks. Police officers in previous studies have expressed a need for more opportunities to take breaks and encouragement from managers or supervisors [37], as some of our previous participants had also noted [15].

Overall, 25% (12/48) of the users reported challenges in initially using shift manager. Some responded that they would continue to use their current apps (eg, Google) for managing their shifts, because of the additional functionality offered by other apps. The ability to add personal notes to the shift manager and to share their weekly or monthly shift pattern with family members was something that users would like to see in an improved iteration. Having these features would cover users who preferred to use something different at the moment.

This feature has become the key central cog that other elements can be integrated with—for example, working in conjunction with the catch-up card function—therefore, the design and use of this function must be simple, effective, and easy to visualize and track. Feedback resulted in the early diary design to input shift being modified and renamed. Currently, there are 6 core shift patterns that have been prepopulated in the app, which users can choose to select. The frequency of catch-up cards was also set according to the shift chosen and included upon client feedback. The ability to view the effect of different factors (food, activity, fluid intake, and sleep) on overall wellness—in conjunction with shift—will prompt police staff and officers to make lifestyle modifications to improve health.

Feedback received from users also provided insights to the developers to improve the shift manager feature and to add a help section to educate beginners about how to add and edit their shift patterns.

### Goal Setting and Catch-Up Cards

Behavior change is likely to play a large role in making an app focusing on health and well-being successful, with the suggestion that increased implementation of behavior change techniques could improve interventions and achieve high levels of user engagement [51]. The goal-setting feature was appreciated by participants when using it within the fluid intake section, and the intention is, once finalized, to integrate this functionality within other domains of the app.

Occupational stress is the main contributor to the risk of police officers and staff developing obesity and increasing the risk of long-term health conditions such as cardiovascular and metabolic diseases. To control this, it is very important to self-manage sleep patterns, take regular breaks, monitor dietary and food habits, regulate water intake, help with smoking cessation, and reduce alcohol consumption. Referring to the study conducted by Voyer [52], nudging offers a choice pattern to users that can make changes in their behavior and how decisions are taken at a personal level to improve health. Therefore, it is considered as a behavioral economic concept in the new world. The study by Kwan et al [53] highlighted that reminding the patient to maintain good habits by sending nudges has the potential to reduce the cost of health care and help patients take long-term control of their health. Self-management empowers users to make decisions in favor of their health, and nudges or reminders keep them more involved and informed in maintaining their health. Nudge theory is a young behavioral economic concept that “influences the behaviour and decision-making of patients through choice architecture” [52]. Nudging is not mandatory; rather, it gives small choices in behavior, at a level that has the potential to influence.

In the initial research requirement gathering, participants mentioned that notifications on the app would help them to perform tasks that they generally forgot, as they did not access the app many times during the day [15]. Catch-up cards were introduced and linked to the shift manager in this version of the app to support and encourage police officers and staff to try and maintain good habits. Catch-up cards are scheduled differently for each shift pattern and rest day to prepare the participants for their next shift. They work as a “nudge” or reminder for the participants to do breathing exercises, sleep on time, complete activity steps, and so on.

### Supplementary Information and Guidance

Previously, some pilot research work was undertaken with project team members on an app to centralize evidence-based nutrition and lifestyle guidance for health care professionals and people living beyond cancer—enabling them to obtain guidance and information and create and track nutrition and activity-related goals [54]. The research work also fed useful reflections into the design of this project. Each domain in the app that requires input from the users has an information section that gives recommendations about how much fluid to take, what the healthy options are, and so on, according to NHS guidelines. Participants found this guiding information to be helpful, as it was easy to refer to in case of query. However, in this study, there were few suggestions received around improving the design to draw more attention to these particular sections.

### Limitations

Although the authors believe that the results from this study can be generalized to other police forces across the country, we acknowledge that there is a limitation of only accessing data from a subsection of 1 regional UK police force, which might have inherent organizational biases toward health and well-being. However, this approach can be expanded to cover more regions in due course. Regarding bias with purposeful sampling—where the belief is that qualitative research should be describing the medium or the norm—the point to underline is that new phenomena are being described; therefore, we needed to purposively select the best examples of what we were interested in. This gave us the clearest cases with the least “noise” or extraneous errors and allowed for the identification of characteristics and boundaries [55]. A further limitation of the study was the greater number of responses from women (32/48, 67%) than those from men when compared with regional UK police workforce numbers as of March 2022, which noted that the gender split was 48% women and 52% men [56].

### Future Studies

The wellness app for the commissioning force has now been subsequently soft launched with funding for the bespoke pattern design agreed upon. The findings from this study are being shared with the developers to analyze the possibilities of implementing them in the next release of the app and have the improved version of the app trialed before its final launch for a wide police population.

#### Organizational Support

In addition to a well-designed mobile app, suitable scaffolded organizational support implemented alongside the eventual release of the app, together with an evaluation of how the app is being used, offers the best chance of producing positive results. This could include encouraging the use of more health-oriented leadership—a style of leadership associated with more well-being and low levels of burnout, depression, and physical complaints among police officers [57].

#### Voice Integration

Emergent technological enhancements (such as Google Voice and Google Assistant) offer opportunities for improved personalized eHealth solutions [58] and increased engagement [59] and adherence [60]. In the area of nutrition, projects are already being conducted, such as the design, development, and evaluation of an Alexa Skill on food and nutrition management for native American patients with diabetes [61]. Similarly, regarding fitness, TandemTrack combines a mobile app and an Alexa skill to support exercise regimens, data capture, feedback, and reminders [62]. Members of this team have previously explored the tentative use of voice-activated speakers or assistants such as Google’s Assistant or Amazon’s Alexa to enable the input of information via voice rather than keyboard [63], which might also assist in making this type of innovation quick to use and therefore more user-friendly.

#### Investigating Other ESP Pathways

These unique issues, the continuing long-term effects of the pandemic on employee health and well-being, and the highlighted gaps in solutions available at present are not only applicable to police staff and officers but also to other ESP—such as firefighters [64,65], paramedics [66,67], and health care professionals [68,69]—and other shift workers. Therefore, the project team is seeking further funding, to explore the development and expansion of this approach to health and well-being issues in other sectors, in addition to the police.

### Conclusions

This study highlights the importance of coparticipation with officers and staff across the entire development cycle, to coproduce a human-centered design methodology to enable the development of a considered and user-centered solution. It demonstrates the need for producing a multifunctional tool rather than focusing purely on an individual element for this user group. It also highlights how linking and being able to track optional, personalized elements of health data against each other, cross-referenced to individual shift patterns, might help to inform and provide users with a chance for reflection and therefore influence behavior change.

## Abbreviations

ESP: emergency service personnel
mHealth: mobile health
MoSCoW: Must Have, Should Have, Could Have, Won’t Have
NHS: National Health Service
SUS: System Usability Scale
